# Love and Lust

**Published:** 1912-10

**Authors:** 


### Love and Lust.

Often much needless and fruitless discussion comes from a failure to have a common understanding of the subject matter considered. Particularly is this true regarding the sexual instinct, because of the hazy knowledge relative to this question. For this reason let us have, at the outset of these discussions, a clear understanding of the meaning of love and its illusions. Not that we shall attempt to define love, as the poet or dreamer, but in its true meaning as it concerns eugenics.

Man is dominated by his sexual passion, whatever else concerns him socially, politically or physically is subservient to this end; it alone can afford the supreme contentment that a normal life demands; it determines his character as a man, his success or failure in life. As Dr. Hall says, "Sex is the largest and most complex, the most important and interesting of all human themes."

Love is sexual passion refined by the soul and controlled through the intellect; born of respect, forbearance, long-suffering and unselfishness. Lust is sexual passion without intellectual refinement—the bent of animal desire to give physical gratification. Husbanded in selfishness, nurtured in sensuality, it ends in the subjugation of the weaker by the stronger.

Love is virtue, the full bloom of the moral instinct; lust is the moral instinct perverted and unrestrained. One is God's purest, best gift to man; the other is the outcome of immoral influences resulting from social conditions.

Love is sacred and indissoluble, and needs no law to compel obedience; lust knows no law, obeys no law and is transitory.

Love ennobles and brightens the home; lust debauches, sickens and despoils it.

When we speak of the sexual life, we must bear in mind this distinction between love and lust. Without love in the land the bonds of society would go asunder. And, just to the extent that lust predominates, deterioration progresses. Love is virtue, and is without price, unsullied and eternal; lust is a question of bargain, and, what is more to the point, it stalks as a symbol of love in our homes, our churches, the reception, the street, and whereever society exists.

It is the strong purpose of eugenics to cultivate virtue and disarm lust; to ennoble the home, and to protect womanhood; to restrict love in the true sense of its meaning and brand sensuality with the deterrent elements of the race. To this end, eugenics advocates the higher education of woman; the higher respect of manhood for womanhood; the industrial emancipation of woman; the elimination of prostitution; the control of venereal diseases; the regulation of marriage; uniform divorce laws, and the equal rights of woman in every human endeavor.

